# Cytoplasmic RNA viruses as potential vehicles for the delivery of therapeutic small RNAs

**DOI:** 10.1186/1743-422X-10-185

**Published:** 2013-06-07

**Authors:** Jose A Usme-Ciro, Natalia Campillo-Pedroza, Fernando Almazán, Juan C Gallego-Gomez

**Affiliations:** 1 Molecular and Translational Medicine Group, Facultad de Medicina, Universidad de Antioquia, Carrera 51D Nº 62 – 29, Piso 3, Edificio Histórico Manuel Uribe Ángel, Medellin, Colombia; 2 Viral Vector Core & Gene Therapy, Neuroscience Group, Sede de Investigación Universitaria – SIU, Universidad de Antioquia, Medellin, Colombia; 3 Department of Molecular and Cell Biology, Centro Nacional de Biotecnología (CNB-CSIC), Campus Universidad Autónoma, Darwin 3, Madrid 28049, Spain

**Keywords:** Cytoplasmic RNA viruses, Gene therapy, microRNAs, Non-canonical pathways, Viral vectors

## Abstract

Viral vectors have become the best option for the delivery of therapeutic genes in conventional and RNA interference-based gene therapies. The current viral vectors for the delivery of small regulatory RNAs are based on DNA viruses and retroviruses/lentiviruses. Cytoplasmic RNA viruses have been excluded as viral vectors for RNAi therapy because of the nuclear localization of the microprocessor complex and the potential degradation of the viral RNA genome during the excision of any virus-encoded pre-microRNAs. However, in the last few years, the presence of several species of small RNAs (e.g., virus-derived small interfering RNAs, virus-derived short RNAs, and unusually small RNAs) in animals and cell cultures that are infected with cytoplasmic RNA viruses has suggested the existence of a non-canonical mechanism of microRNA biogenesis. Several studies have been conducted on the tick-borne encephalitis virus and on the Sindbis virus in which microRNA precursors were artificially incorporated and demonstrated the production of mature microRNAs. The ability of these viruses to recruit Drosha to the cytoplasm during infection resulted in the efficient processing of virus-encoded microRNA without the viral genome entering the nucleus. In this review, we discuss the relevance of these findings with an emphasis on the potential use of cytoplasmic RNA viruses as vehicles for the efficient delivery of therapeutic small RNAs.

## Introduction

Gene therapy has become an important topic in medicine over the last 3 decades [1]. Recombinant DNA technology first allowed the fusion of DNA molecules from different sources [2] and the generation of genetically modified organisms [3]. The possibility of isolating, cloning and expressing genes artificially, the use of different promoters and the delivery of transgenes by recombinant viruses [4-6] have opened a wide spectrum of therapeutic possibilities to treat diseases that are caused by genetic defects. The opportunity of correcting congenital errors by expressing a healthy copy of the disease-causing gene and the use of different vehicles for the delivery of therapeutic genes to the specific tissue or cell type has become a viable treatment option. In conventional gene therapy, which consists of the delivery of transgenes into target cells, *in vivo* (administration directly into the body) and *ex vivo* (target cells are isolated, treated with the therapeutic gene and re-infused or injected into the body) approaches have been used [7] depending on several factors, mainly the localization of the target cell being treated.

Gene therapy is currently being used in clinical trials and viral vectors that are derived from wild type viruses, which have evolved over million years to carry genetic material from one cell to another cell, have been considered the best option for transgene delivery [8]. In addition, after the discovery of RNA interference (RNAi) as a fundamental mechanism of gene silencing in a sequence-specific manner, at the post-transcriptional level [9], the possibility of using the endogenous machinery of this mechanism to artificially down-regulate disease-causing genes, previously untreatable by conventional gene therapy, and the use of viral vectors for the delivery emerged as a new therapeutic tool, and the 2006 Nobel Prize in Physiology or Medicine was awarded to their discoverers [10].

In general, the mechanism of RNAi, and its small interfering RNA (siRNA) pathway mainly involved in the innate defense against viruses and transposable elements [11], starts with the cytoplasmic processing of double-stranded RNAs (dsRNA) by a complex involving the ribonuclease III (RNase III) Dicer, TAR RNA-binding protein (TRBP), and PKR activator (PACT), to produce siRNAs with 20–24 nt in length, and 3′ dinucleotide overhangs. Association of the Argonaute (AGO) with the siRNA duplex promotes the degradation of the siRNA passenger strand and activation of the RNA-induced silencing complex (RISC) containing the siRNA guide strand, which leads to full complementarity, cleavage and degradation of the target mRNA [12]. By the other hand, the natural endogenous biogenesis pathway of miRNAs involved in regulation of physiological and pathological processes such as development, apoptosis, differentiation, and cancer [13], begins with the transcription of a primary miRNA (pri-miRNA) in the nucleus. This pri-miRNA (typically several kilobases long) with stem-loop structures is recognized and cleaved at the stem of the hairpin structure by the Microprocessor complex, which is composed of at least two proteins, the DiGeorge syndrome critical region 8 (DGCR8), and the Ribonuclease III (RNase III) enzyme Drosha [14,15], to generate a miRNA precursor (pre-miRNA) that is approximately 65 nt in length. The pre-miRNA is exported to the cytoplasm by Exportin-5 [16] and cleaved near the terminal loop by a second processing complex involving Dicer, TRBP, and PACT, to generate a miRNA duplex that is approximately 22 nt in length, containing mismatches and 3′ dinucleotide overhangs [12,17,18]. The miRNA duplex associates with RISC, and while the passenger strand is degraded, the guide strand (mature miRNA) remains loaded in this silencing complex to carry out translational repression or exonucleolytic mRNA decay in a sequence-dependent manner [19].

Viral vectors are considered efficient vehicles for RNAi-based therapy because the endogenous machinery can efficiently process the precursor sequences that are included in the viral genomes [20]. These precursors can be directly transcribed from DNA sequences with Pol II or Pol III promoters to produce short hairpin RNAs (shRNAs) or inserted into a viral RNA genome to be processed as functional small RNAs [11]. Alternatively, more efficient processing has been observed for miRNA-adapted shRNAs (shRNAmir), which are second-generation shRNAs exploiting the pri-miRNA environment of a well-characterized family of miRNAs (miR30) [21], and a closely-related strategy where artificial miRNAs precursors with structures that preserve the recognition and processing sites for the RNase III enzymes Drosha and Dicer lead to the production of mature miRNAs with sequence-specific action [22].

Many congenital and acquired diseases respond to gene therapy with viral vectors. For monogenic diseases, the specific restoration of gene function should theoretically result in a cure; however, several problems limit the success. The main problem is that the majority of diseases are complex, and multiple genes and the environment play a role in the clinical manifestations [23]. Additional problems are related to the specific delivery requirements of each disease that could not be filled by available viral vectors. Every disease requires a very specific therapy that could be based on high or low transgene expression, long or short expression periods, repeated administration, or expression in one or multiple tissues or specific cell types. The new frontiers in medicine demand new opportunities for the treatment. Each viral vector has specific advantages, limitations, and range of applications [24]. Therefore, a viral vector may meet the requirements for the treatment of one disease; however, the same vector may not be the best option for the treatment of other diseases.

The available viral vectors that are based on DNA viruses allow for the manipulation of tissue-specific expression by different promoters because transcription is a necessary step before protein expression. A large number of gene switches are available, including hypoxia-, glucose-, tetracycline-, heat-, and radiation-inducible promoters [25]. This feature is not available for vectors that are based on RNA viruses, which lack a DNA phase and therefore a transcription step; however, for RNA viruses it has been assessed by the insertion of targets for tissue-specific miRNAs in the viral vector genome, leading to the silencing of the vector genome or vector-derived transcript in specific cell population expressing the miRNA [26,27]. In addition, RNA virus vectors could be used for specific delivery by changing the envelope epitopes that interact with specific cellular receptors in the same way as reported for DNA viruses [28], or pseudotyping them with envelopes and other structural proteins from closely-related viruses, as shown for alphaviruses and flaviviruses [29-31].

The most frequent limitations in RNAi-based therapy are saturation and toxicity. Saturation is caused by overexpression of artificial small regulatory RNAs, which compete with the endogenous small RNA precursors for the RNAi machinery leading to inhibition of the normal gene regulation by endogenous miRNAs, as demonstrated in *in vitro* and *in vivo* experiments [32]. The main saturation-prone proteins of the RNAi machinery (miRNA and siRNA pathways) at different degrees are Exportin-5, AGO-1 to −4, and RISC. Although Drosha and Dicer saturation is theoretically possible, supporting experiments are needed [33-35]. Cellular and systemic toxicity could be associated with the vector that is used for the delivery and the interaction between the vector and the immune system. Cytoplasmic RNA viruses that are associated with acute infections could be used for therapeutic small RNA delivery because these viruses overcome the main problems that are associated with the saturation of the silencing machinery at the nuclear level [33], Cytoplasmic RNA viruses replicate/transcribe their genomes resulting in high levels of cytoplasmic viral RNA and small regulatory RNAs. The fact that these viruses have been mainly associated with acute infections could diminish the saturation effect associated to high levels and prolonged expression of siRNAs previously observed with viral vectors based on DNA genomes [36]. In addition, the use of alternative non-canonical pathways for miRNA biogenesis (discussed below) could also prevent the saturation problems.

### miRNA-like structures and other small RNAs can be naturally processed from the genome of cytoplasmic RNA viruses in the infected cells

Cytoplasmic RNA viruses belong to several families and have different replication strategies. These viruses have positive- or negative-sense single-stranded RNA genomes. Positive-sense viral RNA inside of the cell will initiate the translation of viral proteins (the replicase complex) before replication of the viral genome. In negative-sense RNA viruses, the viral particle incorporates the RNA-dependent RNA Polymerase (RdRP) to initiate the synthesis of positive strands when delivered into the host cell [37]. In spite of the results of one study, suggesting a small percentage (20%) of flavivirus replication in the nucleus [38], these viruses have been considered cytoplasmic, according to the historically accumulated evidence [39]. Consequently, viral RNA genomes from cytoplasmic viruses should not have the opportunity of meeting the microprocessor complex, which is the first ribonucleoprotein complex that is involved in the miRNA pathway in the nucleus [19]. Exploiting the cellular RNA silencing machinery through the endogenous miRNA pathway as a convenient way to regulate viral and host gene expression was therefore, considered to be exclusive for DNA viruses, and unavailable for RNA viruses replicating in the cytoplasm [40].

Interestingly, several virus-derived small interfering RNAs (viRNAs) that were 21 nt in length and randomly spanned the full-length viral genome were generated by a nonrandom mechanism as the product of a strong antiviral response in *Aedes aegypti* mosquitoes that were infected with the alphavirus Sindbis (SINV), and these viRNAs were associated with a modulation of the pathogenic effect in the mosquitoes [41]. In addition, RNA viruses were shown to generate a diverse range of virus-derived short RNAs (vsRNAs). Multiplexed high-throughput sequencing after infection with 6 different cytoplasmic RNA viruses revealed populations of vsRNAs (10–60 nt long) in cell lines and in the animal model (*Caenorhabditis elegans*) that was known to exploit the RNAi machinery as an antiviral defense. The presence of vsRNAs fluctuated from rare to highly abundant, and several vsRNAs were identified in quantities that were comparable to highly expressed cellular miRNAs [42]. The secondary structure of these vsRNAs is different from miRNA hairpins, which suggests that miRNA processing occurs through alternative non-canonical pathways. The function of these vsRNAs in host and viral processes remains unclear [42]. Interestingly, a comparison of the vsRNA profiles in two mosquito cell lines, *Aedes aegypti* Aag2 and *Aedes albopictus* C6/36 cells after infection with the flaviviruses dengue virus (DENV), the West Nile virus (WNV) and the cell fusing agent virus (CFAV) demonstrated dramatic differences. Aag2 cells were capable of siRNA production, whereas C6/36 cells revealed predominantly longer small RNAs with positive polarity, a result of defective Dicer-2 cleavage of the long dsRNA in these cells [43,44]. RNAi is an important mechanism of antiviral immunity in mosquitoes [45,46]. When RNAi is inefficient (as in the case of C6/36 cells) or specifically inhibited by the B2 protein of the flock house virus (FHV), higher arbovirus replication and mortality were observed *in vitro* and *in vivo*, respectively [47]. In addition, the presence of longer small RNAs in C6/36 cells suggests that other possible small RNA pathways play a role in antiviral immunity and other active processes in the host-virus interaction.

Two new classes of viral small RNAs, ping-pong PIWI-interacting RNA-like (ping-pong piRNA-like) small RNAs, which are approximately 23–30 nt in length, and the unusually small RNAs (usRNAs), which are approximately 13–19 nt in length, were identified during the characterization of the small RNA profile of DENV-infected mosquitoes [48,49] and mosquito cells that were infected with the SINV and the bunyavirus La Crosse virus (LACV) [50].

Recently, a mature miRNA-like structure (KUN-miR-1), which was derived from the 3′ terminal stem-loop (3′SL) of the WNV, was detected in abundance in mosquito cells after infection with the Kunjin strain of the virus (WNV_KUN_) [51]. KUN-miR-1 was found to target mosquito GATA4 mRNA, which leads to up-regulation of this gene in the cells. GATA4 silencing was correlated with reduced virus replication, whereas the up-regulation of GATA4 by KUN-miR-1 was correlated with enhanced virus replication in mosquito cells [51].

The evidence of the viRNAs, vsRNAs, ping-pong piRNA-like, usRNAs and functional mature miRNA-like structures in cytoplasmic RNA virus-infected cells clearly suggest the interaction of these viral genomes with components of the non-canonical pathways of small RNA processing of the host cells.

### The role of non-canonical pathways of miRNA biogenesis in the processing of viral small RNAs

The analysis of high-throughput sequencing data from many taxa have revealed subclasses of miRNA species and other related small RNAs that are produced by alternative non-canonical Drosha- or Dicer-independent miRNA biogenesis pathways [52]. Mirtrons are the main representatives of the Drosha-independent pathways [53]. Mirtrons are short introns that are initially processed by the spliceosome to generate pre-miRNAs, which are subsequently exported from the nucleus by Exportin-5, processed by Dicer and loaded in the RISC to carry out silencing [54]. Functional miRNA-like small RNAs that are derived from small nucleolar RNAs (snoRNAs), which are a class of non-coding RNAs that are localized to the nucleolus, are processed in a Drosha/DGCR8-independent and Dicer-dependent manner [55] in animals, plants and yeast [56], and constitute the snoRNA-derived pathway [57]. Additional Drosha-independent pathways, such as the tRNA-derived [58], endogenous shRNA-derived [59], tRNase Z-dependent [60], and endogenous siRNAs (endo-siRNAs) [59] pathways, have been recently described (See Miyoshi et al. [57] for an extended review). Non-canonical miRNA biogenesis can also occur in a Dicer-independent manner, such as in the Splicing-independent mirtron-like miRNAs (Simtrons) pathway in mammals, which involves Drosha but is independent of DGCR8, Exportin-5, Dicer, and AGO-2 [61].

Little is known about the role of these non-canonical pathways of miRNA biogenesis in modulating the virus-host relationship. Although, small regulatory RNAs have been hypothesized to modulate the antiviral immunity, and to participate in regulation of cellular processes to increase viral replication [62], several questions emerge from the production of viral small RNAs in cytoplasmic RNA virus-infected cells ¿What is the specific biogenesis pathway allowing their generation? If they are the product of an evolutionarily conserved mechanism ¿What is the functional role of these small RNAs in modulating host cell processes? ¿Do they participate in virus replication? ¿Do they increase pathogenicity? ¿Are the small virus-derived RNAs byproducts of the antiviral response? Some of these questions have been only partially answered and require further investigation.

### Cytoplasmic RNA viruses as vehicles for the delivery of small RNAs that target disease-causing genes

Viral miRNAs have been identified in a growing number of DNA viruses, including herpesviruses, polyomaviruses, adenoviruses, and ascoviruses [62]. Although two papers have described the identification of miRNAs in the human immunodeficiency virus-1 (HIV-1), and in the bovine leukemia virus (BLV) [63,64], which are RNA viruses belonging to the *Retroviridae* family, with a nuclear DNA phase (provirus) in their replication cycles, for other RNA viruses, miRNAs were hypothesized to be absent because of the potential degradation of the viral RNA genome during the excision of virus-encoded pre-miRNA [40]. To test this hypothesis, a cellular neuron-specific pri-miRNA (pri-miR-124) was incorporated into the genome of the influenza A virus (IAV), which is a nuclear RNA virus without a DNA phase during its replication cycle [65]. The production of pre-miRNA-124 (pre-miR-124) and functional mature miRNA-124 (miR-124) was demonstrated without a detrimental effect on viral replication or genome stability, which could be the result of Nuclear Export Protein (NEP) mRNA rather than viral genomic RNA being the favorable substrate for Drosha-cleavage [65].

The potential degradation of the viral RNA genome during viral miRNA biogenesis and the presumed absence of Drosha in the cytoplasm during pri-miRNA processing suggest a low probability of finding virus-encoded miRNAs in cytoplasmic RNA viruses [40]. This supposition was refuted in studies that used two different cytoplasmic RNA viruses, the tick-borne encephalitis virus (TBEV) [66], and the SINV [67], independently. miR-BART2, which is a miRNA precursor from the Epstein-Barr virus (EBV) [68], was incorporated into the 3′ non-coding region of the TBEV genome, and using a dual-luciferase reporter assay, the generation of the functional miRNA was demonstrated without impairing of viral RNA replication [66]. In the same study, the role of Drosha in the formation of the artificial miRNA by flaviviruses was suggested [66]. Additionally, pri-miR-124 was incorporated into the viral genome of the SINV and able to produce pre-miR-124 and miR-124 in a DGCR8-independent, exportin-5-independent and Dicer-dependent manner [67]. Interestingly, in a recent study, localization of pri-miR-124 to the cytoplasm during miRNA biogenesis was demonstrated after infection with rSINV124 (the SINV that carries pri-miR-124), and was also associated to a strong redistribution of Drosha to the cytoplasm without a negative impact on the endogenous miRNA profile [69]. Altogether, the evidence suggest a novel pathway for cytoplasmic pri-miR-124 processing in a Drosha-dependent manner [69]. In the same study, loss of DGCR8 resulted in the accumulation of pre-miR-124 and low levels of mature miR-124, suggesting that DGCR8 could determine the accuracy of the Drosha-mediated cleavage, leading to optimal pre-miR-124 for Dicer cleavage [69]. Notably, these findings suggest a function of Drosha in the cytoplasm for processing of pri-miRNA structures and supports previously obtained data of miRNA processing from genetically-engineered viruses [66,67].

**Figure 1:**
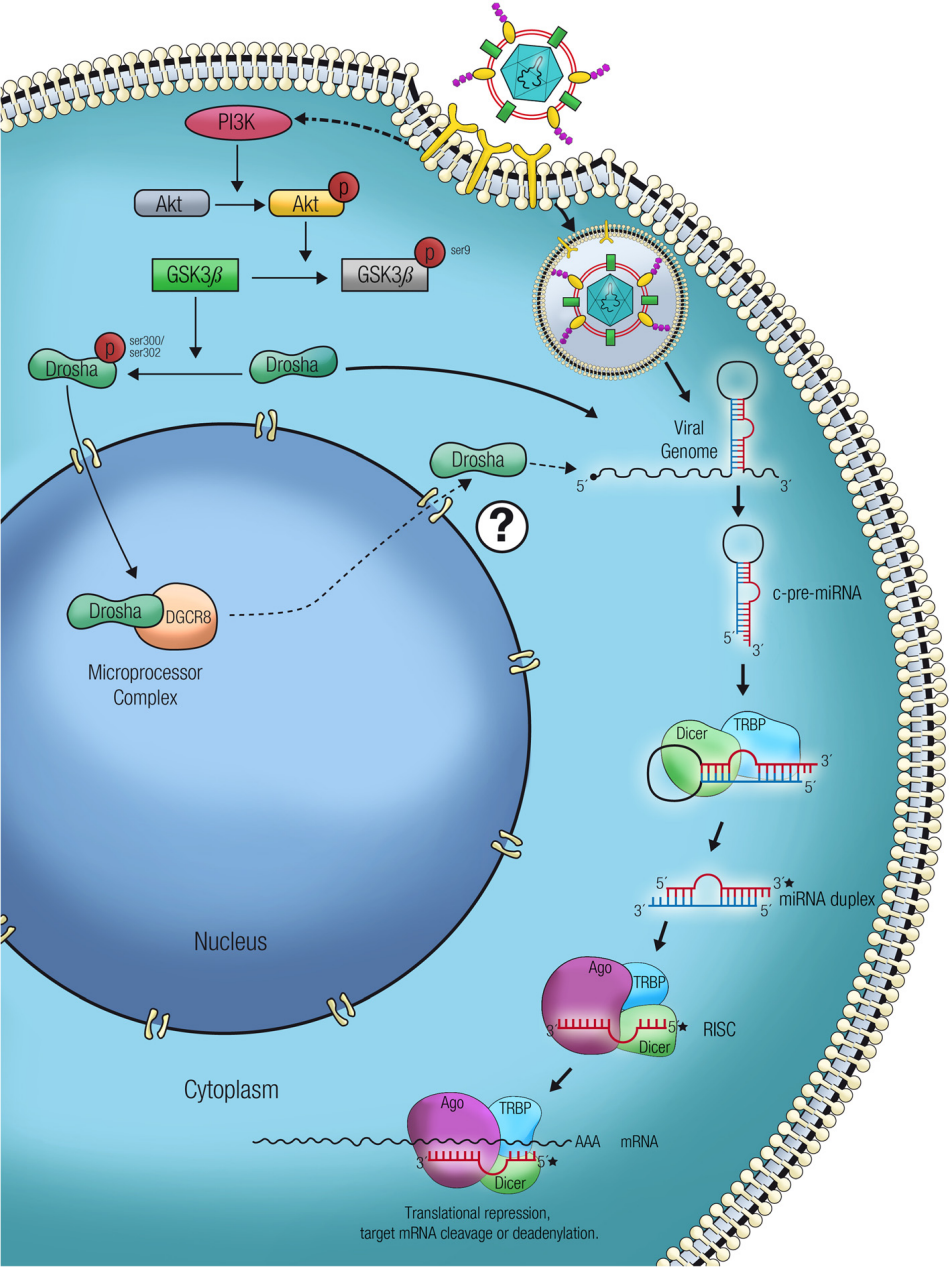
**Proposed model for non-canonical cytoplasmic processing of virus-encoded miRNA-like structures in a Drosha-dependent manner.** The PI3K/Akt pathway is known to be activated early during flavivirus infection, which in turn leads to the GSK3β inactivation by phosphorylation at Serine9. GSK3β is the kinase responsible of Drosha phosphorylation at residues Serine300 or Serine302, which is required for its nuclear localization. In the absence of active GSK3β, the unphosphorylated Drosha should accumulate in the cytoplasm where it should be available to start a non-canonical cytoplasmic miRNA biogenesis pathway. To establish if the absence of Drosha phosphorylation is enough for explaining its resulting cytoplasmic pattern, or the nuclear Drosha is actively relocalized to the cytoplasm remains to be demonstrated.

On the one hand, the nuclear localization of Drosha depends on its phosphorylation at residues Serine300 and/or Serine302 [70], where glycogen synthase kinase 3 beta (GSK3β) is indispensable [71]. On the other hand, Akt inhibits GSK3β by phosphorylation at residue Serine9 [72] and Akt phosphorylation and activation in a phosphatidylinositol 3-kinase (PI3K)- and lipid raft formation-dependent manner as a response to viral infections with cytoplasmic RNA viruses has been demonstrated (e.g., the DENV and the JEV) [73]. Deciphering the precise cellular signaling that is responsible for Drosha redistribution during viral infections and determining whether the dramatic relocalization is associated with an active protein export process or the result of cytoplasmic accumulation of non-phosphorylated Drosha, could explain the processing of miRNA-like structures in cytoplasmic viral genomes by non-canonical pathways. The absence of a negative impact of Drosha redistribution on the cellular miRNA landscape [69] suggests the presence of a functional nuclear microprocessor complex in which phosphorylated Drosha in the nucleus corresponds to the remaining protein from an incomplete turnover, despite the apparent absence in the nucleus as shown by immunofluorescence [69]. A proposed model of cell signaling that is involved in Drosha redistribution during viral infection, which leads to the non-canonical cytoplasmic processing of virus-encoded miRNA-like structures, is depicted in Figure 1.

**Figure 2:**
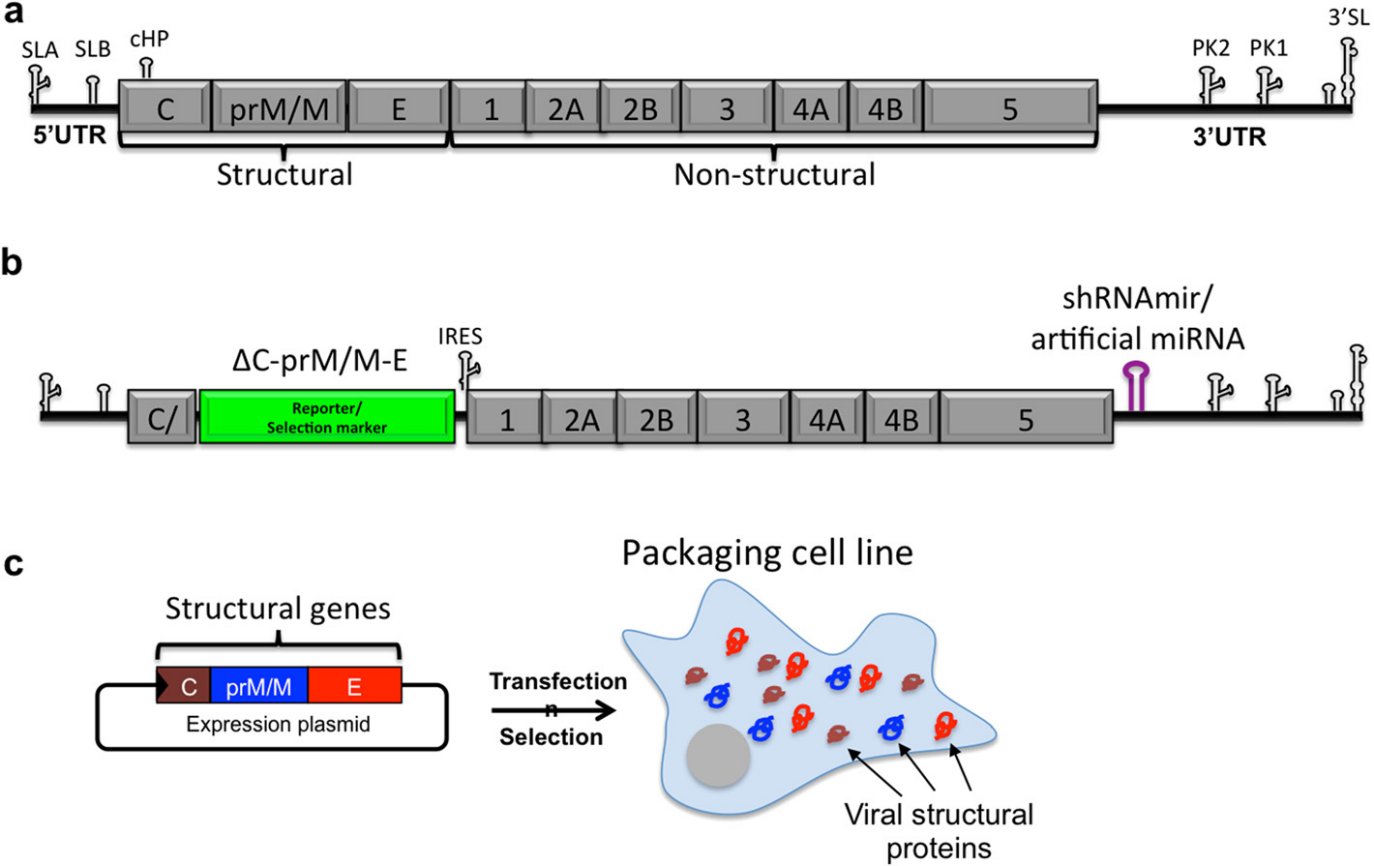
**Turning a flavivirus into a viral vector for therapeutic small RNA delivery. a**) The flavivirus genome consists of a 5′ untranslated region (5′UTR), followed by an open reading frame (encoding the structural and non-structural proteins as a polyprotein), and finishing with the 3′UTR. Some *cis*-acting RNA secondary structures that participate in virus replication and translation are depicted. **b**) The first step during generation of a flavivirus-based viral vector is the construction of a flavivirus replicon by reverse genetics, in which the excision/replacement of the structural genes by the reporter/selectable marker (green), and the insertion of the DNA encoding a shRNAmir or artificial miRNA precursor in the hypervariable region of the 3′UTR can be performed. **c**) Viral structural genes can be expressed in *trans* from an expression plasmid or a packaging cell line generated by selection of the transfected cells, ensuring biosafety by allowing replication but avoiding propagation of the flavivirus vector after transduction of the target cells.

The ability of cytoplasmic RNA viruses to efficiently deliver mature miRNAs into cells has presented a new possibility of engineering RNA viruses as vectors for the specific silencing of disease-causing genes and as vectors against viral diseases [74]. For example, transforming flaviviruses into viral vectors for small regulatory RNA delivery could be assessed by eliminating the viral structural genes from the vector genome, and supplying these genes in *trans* for the packaging of new replication-competent and propagation-deficient viral vectors, and introducing the pri-miRNA-like sequence into the variable region of the 3′UTR as previously described [66] (Figure 2). Importantly, the efficient *in vivo* delivery of mature miRNAs that are artificially incorporated into the viral genomes of positive-sense (SINV) and negative-sense (vesicular stomatitis virus-VSV) cytoplasmic RNA viruses in a wide range of tissues strongly supports the use of these viruses as vehicles for the delivery of therapeutic small RNAs by exploiting the endogenous machinery of non-canonical miRNA biogenesis [74,75].

Gene therapists have called for novel viral and non-viral vectors with different properties that satisfy the specific requirements of potentially tractable diseases [1]. Several clinical trials that use RNAi therapy are currently being conducted [8]; however, new and improved viral vectors are needed to maximize the safety and efficacy [32,76]. A comparison of the different features, advantages and drawbacks, between the main cytoplasmic RNA viruses and some of the RNA and DNA viruses that are available or proposed for RNAi-based therapy is showed in Table 1. Members of the *Alphavirus*, *Flavivirus*, and *Vesiculovirus* genera, clearly emerge as attractive vectors, as demonstrated by the growing experimental evidence showing their ability to process natural or artificial miRNA-like structures from their viral RNAs during infection [51,66,67,74,75]. The genome of these viruses is small (11–12 kb), allowing easy manipulation, cloning, and sequencing. They have high expression levels [77,78], cytoplasmic replication without insertion into the host genome, can be non-cytopathic [79,80], can be packaged into virus-like replicon particles (VLPs or VRPs) [81,82], and could be the ideal vectors for specific applications.

**Table 1:** Comparison between the cytoplasmic RNA viruses and the currently used RNA and DNA viruses for RNAi-based therapy

**Genus**	**Main representative**	**Genome(sense)**	**Genome size (Kb)**	**Immuno-genicity**	**Expression level**	**Genomic insertion**	**Time of expression**	**Advantages**	**Limitations**
*Alphavirus*	*Sindbis virus*	ssRNA (+)	12	High	High	No	Short-term	- Cytoplasmic replication allowing high expression levels and potential non-canonical processing of artificial miRNAs.	Toxicity due to viral replication (non-cytopathic vectors overcome this limitation)
								- Apoptosis induction, they can be considered in Cancer gene therapy applications.	
								- Infect neurons in primary and cell lines, could be good candidates for gene therapy in Central Nervous System (CNS)	
*Flavivirus*	*West Nile virus*, *Tick-borne encephalitis virus*	ssRNA (+)	11	Medium	High	No	Short-term	- Cytoplasmic replication allowing high expression levels and potential non-canonical processing of artificial miRNAs.	Toxicity (non-cytopathic vectors available Pre–existing immunity mainly in (sub)tropical countries
								- Genetic structure allow easy manipulation	
*Vesiculovirus*	*Vesicular stomatitis virus*	ssRNA (-)	11	High	High	No	Short-term	- Cytoplasmic replication allowing high expression levels and potential non-canonical processing of artificial miRNAs.	Very sensitive to the antiviral action of interferon
								- Apoptosis induction, they can be considered in cancer therapy	
*Lentivirus*	*Human immunodeficiency virus* type 1	ssRNA (+)	8	Low	High	Yes	Long-term	Persistent gene transfer in most tissues	Integration might induce oncogenesis
*Dependovirus*	*Adeno-associated virus* serotype 2	ssDNA	<5	Low	High	Yes	Long-term	Non-pathogenic parental viruses	Integration might induce oncogenesis
*Herpesvirus*	*Herpes simplex virus* type 1	dsDNA	150	High	High	No	Short-,Medium-term	Well suited as oncolytic vector and CNS applications (retrograde axonal transport)	Risk of recombination with latently herpes simplex virus-infected cells

Therefore, cytoplasmic RNA viruses constitute the newest alternative for RNAi therapy with a promising future as demonstrated by *in vivo* experiments [75].

## Conclusions

In light of recent findings, cytoplasmic RNA viruses have emerged as potential vehicles for the efficient delivery of small regulatory RNAs inside of cells for therapeutic purposes. The existence of non-canonical pathways of miRNA biogenesis, the redistribution of Drosha to the cytoplasm in infected cells, and the various small RNA species that are found in infected cells and animals suggests that these viruses exploit or interact with several pathways during the course of infection. It is necessary to establish whether Drosha redistribution is a common cellular effect among cytoplasmic RNA virus infections and which proteins are involved in the cellular signaling. The mechanism that is involved in viral non-canonical miRNA processing has not been fully elucidated; however, the results of *in vivo* experiments support the usefulness of this mechanism for therapeutic purposes. These vectors represent a novel tool that could potentially be added to the battery of available vectors that meet the specific therapeutic requirements for every tractable disease.

## Abbreviations

3′SL: 3′ terminal stem–loop; cHP: Capsid hairpin; DGCR8: DiGeorge syndrome critical region 8; miRNA: microRNA; PK1: Pseudoknot 1; PK2: Pseudoknot 2; pri-miRNA: Primary microRNA; RNAi: RNA interference; sfRNA: Subgenomic flavivirus RNA; SINV: Sindbis virus; SLA: Stem-loop A; SLB: Stem-loop B; TBEV: Tick borne encephalitis virus; vsRNAs: Virus-derived short RNAs.

## Competing interests

The authors declare that they have no competing interests.

## Authors’ contributions

JAUC conceived the study and drafted the manuscript. NCP searched the literature and helped in manuscript write-up. FA critically reviewed the manuscript. JCGG conceived the study and critically reviewed the manuscript. All authors read and approved the final manuscript.

## Authors’ information

JAUC (Biologist, MSc in molecular evolution of viruses), PhD candidate in molecular biology and virology); NCP (Biologist, MSc student in basic biomedical sciences); FA (PhD in Molecular and Cellular Biology); JCGG (Biologist, PhD in Molecular and Cellular Biology).
